# Successful implantation after reducing matrix metalloproteinase activity in the uterine cavity

**DOI:** 10.1186/1477-7827-11-37

**Published:** 2013-05-11

**Authors:** Noriko Yoshii, Toshio Hamatani, Noboru Inagaki, Takeshi Hosaka, Osamu Inoue, Mitsutoshi Yamada, Rei Machiya, Yasunori Yoshimura, Yasushi Odawara

**Affiliations:** 1 Fertility Clinic Tokyo, 2-11-16 Ebisu-Nishi Shibuya-ku, Tokyo 150-0021, Japan; 2 Department of Obstetrics and Gynecology, Keio University School of Medicine, 35 Shinanomachi Shinjuku-ku, Tokyo 160-8582, Japan; 3 Saint Women’s Clinic, 9–1 Higashi-Takasagocho, Urawa-ku, Saitama 330-0055, Japan

**Keywords:** Recurrent implantation failure (RIF), Matrix metalloproteinase (MMP), Uterine inflammation, Endometritis, Uterine flushing

## Abstract

**Background:**

Recently, the concept of recurrent implantation failure (RIF) in assisted reproductive technology has been enlarged. Chronic uterine inflammation is a known cause of implantation failure and is associated with high matrix metalloproteinase (MMP) activity in uterine cavity flushing. MMP activity of women with RIF has been reported to be higher than that of fertile women. In the present retrospective study we evaluated the efficacy of treatment for high MMP activity in the uterine cavity of patients with RIF.

**Methods:**

Of the 597 patients recruited to the study, 360 patients underwent MMP measurements and 237 patients did not (control group). All patients had failed to become pregnant, despite at least two transfers of good-quality embryos. Gelatinase MMP-2 and MMP-9 activity in uterine flushing fluid was detected by enzymology (MMP test). All samples were classified into two groups (positive or negative) based on the intensity of the bands on the enzyme zymogram, which represents the degree of MMP activity. Patients who tested positive on the initial test were treated for 2 weeks with a quinolone antibiotic and a corticosteroid, and subsequently underwent a second MMP test. Negative results on the second MMP tests after treatment and subsequent rates of pregnancy and miscarriage were used to evaluate the efficacy of treatment. Data were analyzed by the Mann–Whitney *U*-test and the chi-square test.

**Results:**

Of the patients who underwent the MMP test, 15.6% had positive results (high MMP activity). After treatment, 89.3% of patients had negative results on the second MMP test. These patients had a significantly better pregnancy rate (42.0%) than the control group (26.6%), as well as a lower miscarriage rate (28.5% vs 36.5%, respectively).

**Conclusions:**

A 2-week course of antibiotics and corticosteroids effectively improves the uterine environment underlying RIF by reducing MMP activity.

## Background

In recent decades, many infertile couples, who otherwise would not have been able to have children, have benefited considerably from assisted reproductive technology (ART). However, despite the selection of morphologically good embryos for transfer, there are still some women who fail to become pregnant. Recently, the concept of recurrent implantation failure (RIF) has emerged because the implantation process remains the least understood step in ART. It is known that the success of embryo implantation depends primarily on the quality of the embryos transferred. Nonetheless, embryo quality alone cannot fully explain all cases of RIF, particularly in young women, whose embryos should be of good quality [1,2].

Uterine receptivity is another factor crucial for successful implantation. Numerous studies have sought to identify clinically useful markers to indicate a receptive uterine state. However, it is difficult to select useful molecular biomarkers associated with reproductive failure because endometrial receptivity is governed by the expression of a complex network of mediators, including cell adhesion molecules (e.g. integrins and cadherins), prostaglandins, and cytokines (e.g. leukemia inhibitory factor [LIF] and epithelial growth factor [EGF]).

Chronic endometrial inflammation is a known cause of implantation failure. Indeed, chronic and mild inflammations are often seen in unfavorable uterine conditions other than infectious diseases. For example, the number of macrophages and T cells, which mainly secrete inflammatory cytokines, is increased in the endometria of women with an implanted intrauterine device (IUD) [3], and the contraceptive activity of the IUD is brought about by the maintenance of this environment, which is unfavorable for implantation. As another example, implantation rates following IVF-ET are lower in patients with hydrosalpinx compared with rates in those with unexplained or male sterility because of the leakage of hydrosalpinx fluid (HF), which contains leukocytes and inflammatory cytokines such as interleukin (IL)-8, IL-12, IL-α, and tumor necrosis factor (TNF)-α, contributes to the deterioration of the intrauterine environment, leading to implantation failure [4-6]. Thus, chronic and mild uterine inflammation induces inflammatory cells and alters the expression and subtle balance of various molecules, without any overt clinical symptoms, contributing to implantation failure.

In the present study, we focused on matrix metalloproteinases (MMPs) as candidate biomarkers of chronic and mild uterine inflammation. The MMPs are a family of zinc-dependent proteolytic enzymes that play a major role in the degradation and rebuilding of the extracellular matrix (ECM), as well as in cell migration. Abnormal MMP expression is involved in the pathogenesis of autoimmune diseases, cancer, endometriosis, and other inflammatory diseases [7], although MMPs are also crucial for the normal physiology of the reproductive system [8-10]. Of the >20 types of MMPs, MMP-2 and MMP-9 are representative gelatinases that are present in human uterine endometrial stroma [11,12]. Both MMP-2 and MMP-9 play an integral role in human embryo implantation and are the main rate-limiting enzymes in ECM remodeling during implantation. However, MMP-2 and MMP-9 hyperactivity is associated with an unfavorable uterine environment, similar to that seen with endometrial inflammation, because successful implantation depends on a tight balance between the activation and inhibition of MMPs [13,14]. Inagaki *et al.* reported significantly higher intrauterine MMP-2 and MMP-9 activity, as well as IL-β levels, in women with RIF compared with fertile women [15]. These observations suggest that MMP activity is a sensitive biomarker of an unfavorable uterine environment for implantation, such as chronic endometritis.

Accordingly, we hypothesized that treatment of the chronic intra-uterine inflammation underlying excessive MMP activity may improve uterine receptivity for transferred embryos. Therefore, in the present retrospective study, we evaluated the efficacy of anti-inflammatory treatment, comprising of corticosteroids and antibiotics, in reducing high MMP activity in women with RIF and improving ART outcomes.

## Methods

### Patients

**Figure 1:**
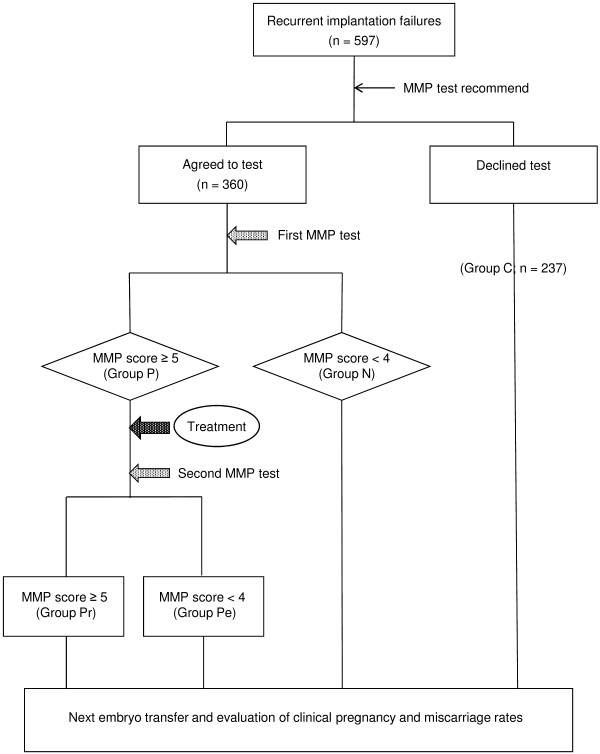
**Flow chart of the study design.** In all, 597 patients with RIF were recruited and divided into two groups based on whether or not they agreed to undergo the matrix metalloproteinase (MMP) test. Patients with MMP scores ≥5 were deemed to have a positive response (Group P) and were treated with a corticosteroid and an antibiotic for 2 weeks. Patients with low MMP scores (<5) were determined as negative (Group N). Patients in Group P were given a second MMP test after the treatment and were divided into two subgroups: Group P_r_ (patients who had again positive results and were considered as resistant to the treatment) and Group P_e_ (patients in whom the treatment was effective). Patients who did not undergo an MMP test comprised the control group (Group C). Patients in all groups underwent another embryo transfer procedure and the outcomes were compared among the four groups.

The present study was performed between July 2006 and June 2011 on 597 RIF patients (see Figure 1). All patients had undergone at least two transfers of good-quality cleavage or blastocyst-stage embryos, but had failed to become pregnant. The definition of RIF varies among different infertility clinics [16]. For example, the 2005 ESHRE PGD Consortium proposed that RIF be defined as “>3 embryo transfers with high-quality embryos or the transfer of ≥10 embryos in multiple transfers; exact numbers to be determined by each centre” [17]. In our clinic, almost 80% of embryos transferred in recent years have been blastocysts (Table 1), which has resulted in better pregnancy outcomes. In 2005, the clinical pregnancy rate at first embryo transfer in our clinic was 39.7% following the transfer of at least one good-quality embryo; the clinical pregnancy rate following a second embryo transfer (after failure of the first) was also good at 37.1%. Based on these data, in our clinic most patients whose embryos are of good quality are likely get pregnant after two embryo transfers. Therefore, in the present study we defined RIF as “≥2 embryo transfers with high-quality embryos” and recommended patients undergo an MMP test after two failed embryo transfers. In the present study, “good-quality” embryos were defined as those that were higher than Grade BB according to Gardner’s criteria [18] or higher than Grade G2 according to Veeck’s criteria [19]. In each case, one or two embryos were selected for transfer. Both fresh and vitrified–thawed embryo transfer cycles were included in the present study. A clinical pregnancy was confirmed by transvaginal ultrasound observation of a gestational sac.

**Table 1:** Clinical characteristics of two patients groups, MMP test group and control group

	**Test group**	**Control group**	***P*-value**
*No. patients*	360	237	
*Age (years)*	38.1 ± 3.6	37.9 ± 3.6	NS
*Prior to intervention*			
*Day 3 serum FSH (mIU/L)*	9.67 ± 3.09	9.68 ± 3.50	NS
*Total no. transfers*	2.28	2.14	NS
*Total no. embryos transferred*	3.27	3.11	NS
*% Blastocysts*	76.7	78.4	NS
*No. embryos transferred during the last procedure*	1.66	1.69	NS

Where appropriate, data are given as the mean ± SD.

**Figure 2:**
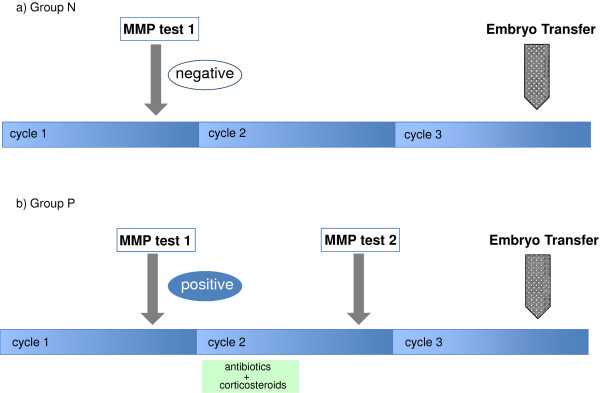
**A schedule of the MMP tests, the medication and the next embryo transfer.** According to the results of the first MMP test, all the patients were divided into two groups, Group N (Panel **a**) and Group P (Panel **b**). Group P patients who had shown a positive result had a two-week treatment composed of antibiotics and corticosteroids at the next proliferative phase (panel **b**). After the treatment, Group P patients had the 2nd MMP test at the mid luteal phase of the same cycle, whereas Group N patients did not have the 2nd test (Panel **a**). In the subsequent cycle (the next cycle after the completion of the treatment), Group P patients had the next embryo transfers.

The 597 patients included in the present study were divided into two groups based on whether they had undergone the MMP test, which measured MMP-2 and MMP-9 activity in uterine flushing fluid: 360 patients underwent the MMP test after repeated unsuccessful transfers, whereas the remaining 237 did not (Group C; control group). Of the patients who underwent the MMP test (see below), those with higher MMP activity (score ≥5; Group P) were treated for 2 weeks with an oral corticosteroid plus an antibiotic. After treatment, the patients underwent a second MMP test to evaluate the efficacy of treatment (see Figure 2). Based on the results of the second MMP test, Group P patients were further subdivided into those who were medication resistant (Group Pr) and those in whom the treatment was effective (Group Pe). All patients then underwent the next round of embryo transfers, with the outcomes evaluated in terms of the clinical pregnancy and miscarriage rates.

Patients ≥43 years of age were excluded from this study even if a good-quality embryo was obtained. All patients underwent transvaginal ultrasound scanning and hysterosalpingography, and those with findings suggestive of macropolyps, submucosal myomas, or anomalies of the uterine cavity were examined further by hysteroscopy. To confirm diagnoses of leiomyoma, adenomyosis, or other organic dysfunctions, magnetic resonance imaging (MRI) was often used. Endometrial biopsies were performed only in those women with abnormal genital bleeding or with findings of uterine endometrial thickening (≥15 mm). Patients diagnosed as having uterine complications, such as endometrial hyperplasia, leiomyoma, adenomyosis, or endometrial polyps, were excluded from the present study, because a previous report has already demonstrated that uterine flushing samples from women with uterine complications have higher MMP activity than samples from women without complications [20]. Patients with uterine cavity anomalies were also excluded from the study.

The present study was approved by the Ethics Committees of the Japanese Institution for Standardizing Assisted Reproductive Technology. Samples were collected only from those patients who provided informed consent.

### Uterine flushing fluid

Samples were collected at the mid luteal phase, the fifth to seventh day post-ovulation, which is considered as an implantation window. The method used to irrigate the uterine cavity was as described previously [15,20-22], using an 8 Fr Foley catheter and a syringe filled with 5 mL saline solution. The catheter was first inserted into the uterine cavity through the cervix and then connected to the syringe. Subsequently, ≤5 mL saline solution was injected into the cavity and aspirated immediately without contamination by the vaginal and cervical fluids. The samples collected were centrifuged at 1000*g* for 10 min to remove blood corpuscles, large-sized protein molecules, and other impurities. The supernatant was collected and stored at −80°C until use.

### Gelatin zymography for MMP-2 and MMP-9 activity

Gelatin zymography was performed according to methods of Fridman *et al.*[23], with the slight modifications of Kleiner and Stetler-Stevenson [21]. Gelatin (final concentration 1 mg/mL) was incorporated into the running polyacrylamide gel containing 10% acrylamide (Bio-Rad, CA,USA), 25% Tris buffer (1.5 mol/L, pH 8.8), 0.4% sodium dodecyl sulfate (SDS; Sigma-Aldrich Japan, Tokyo, Japan), 0.3% ammonium peroxodisulfate (APS; Sigma-Aldrich Japan, Tokyo, Japan), and 0.1% *N*,*N*,*N*,*N*-tetramethylethylendiamine (TEMED; Sigma-Aldrich Japan, Tokyo, Japan). The stacking gel contained 2% acrylamide, 25% Tris buffer (0.5 M Tris, pH 6.5), 0.4% SDS, 0.4% APS, and 0.1% TEMED, and was put on the top of the running gel. The same volume of sample buffer, consisting of 17.5% SDS, 7% sucrose, and bromophenol blue, was added to each sample. Then, 10 μL of each sample was loaded into individual wells and proteins were electrophoresed for approximately 1 h at 200 V. After electrophoresis, the gels were washed five times for 5 min each time in a Tris-based solution consisting of 3% Triton X-100 (Wako, Osaka, Japan). The gels were then washed three times and incubated for 48 h at 37°C in a solution containing 5.8% Tris–HCl, 1.7% Tris base, 0.1% NaN_3_, 0.7% CaCl_2_2H_2_O, and 5% of 100 μM ZnCl_2_. After incubation, the gels were stained for 6 h with 0.1% Coomassie brilliant blue.

The presence of gelatinases (MMP-2 and MMP-9) was confirmed by their inhibition using EDTA and *o*-phenanthroline. Other MMPs that can be detected by casein rather than gelatin zymography (e.g. MMP-1, MMP-3, and MMP-7) were not detected in diluted fluid from the uterine cavity.

### Quantitation of MMPs on zymograms

All samples were diluted 20-fold before being run on the gels. When clear bands were seen against the blue gel background, gelatinase activity was determined on the basis of the intensity of those bands. Individual bands were identified on the gels for proMMP-2, active MMP-2, proMMP-9, active MMP-9, and dimeric MMP-9. Zymograms can be quantitated by densitometric analysis, but this is accurate only at picogram levels of MMPs when the bands on the zymograms are very faint. Because of the high gelatinase activity in uterine flushing fluid, an alternative semiquantitative technique was used to assess gelatinase activity in this study, rather than densitometric measurement which can underestimate the intensity of strong bands [24]. Two independent observers scored the intensity of each band by visual inspection from 0 (no band) to 5 (very strong). A total MMP score for each sample was achieved by adding the scores for each band in that sample. To confirm the reliability of the method of evaluation, we performed preliminary studies in which three observers independently scored the intensity of bands in >100 samples. The results indicated a concordance rate of total scores ≥95%. In addition, the incidence of a ≥2 difference in total scores among the three observers was ≤2%; the incidence of a ≥3 difference in total scores was 0%. As a standard, we used the supernatant of BHK9 breast carcinoma cell culture in the present study.

### MMP scoring system

A total MMP score was represented from 0 to 25 [24]. Since mean total MMP score of RIF patients was demonstrated as 5.0 ± 3.1 [15], samples with a score >4 were defined as having high MMP activity and as a positive result on the MMP test. Samples with scores of 0–4 were deemed negative. All women who tested positive were recommended to start treatment with an oral corticosteroid (prednisolone 10 mg/day; Predonisolone®; TAKEDA, Osaka, Japan) and a quinolone antibiotic (ofloxacin 300 mg/day; Tarivid®; DAIICHI SANKYO, Tokyo, Japan). Treatment lasted for 2 weeks. After the treatment period, samples of uterine flushing fluid were collected at the next luteal phase and the MMP test repeated.

### Statistical analyses

The outcomes of transfers in the different groups were evaluated by comparing the clinical pregnancy and miscarriage rates. Data were analyzed by the Mann–Whitney *U*-test and the *χ*^2^ test. All analyses were performed using SPSS version 19 (IBM Japan, Tokyo, Japan). *P <* 0.05 was considered to be statistically significantly different.

## Results

The 597 RIF patients in the present study were divided into two groups depending on whether they agreed to undergo an MMP test after receiving a comprehensive explanation of the procedure. The characteristics of patients in both groups are summarized in Table 1. There were no significant differences in age, Day 3 serum FSH levels, total number of transfers, the number of embryos transferred, or the percentage of blastocysts (not cleavage-stage embryos) that developed from the transferred embryos between the two groups before the treatment intervention.

**Figure 3:**
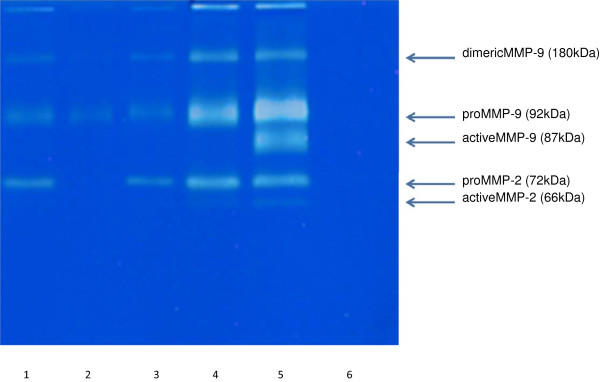
**A representative image of a gelatin zymogram used for MMP scoring in a MMP test.** The lanes represent uterine fluid samples from individual patients with recurrent implantation failure (RIF). Lanes 1, 2, 3, 4 and 6 were scored 4, 2, 4, 8 and 0, respectively. Lane 5 shows a result from the standard used in the present study (supernatant of BHK9 breast carcinoma cell culture). Details of the scoring system are described in the Methods section.

**Figure 4:**
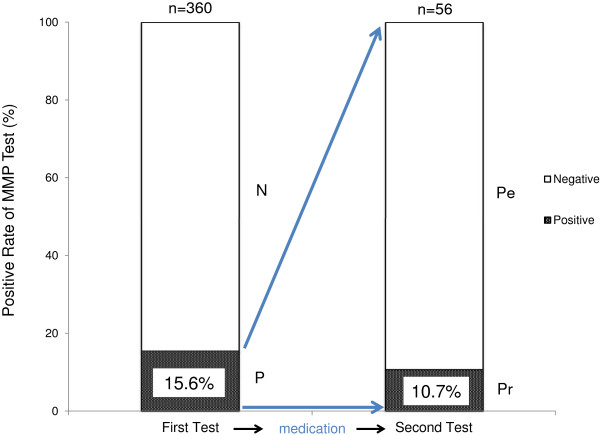
**The MMP test results before and after the treatment of patients with RIF.** Prior to the treatment, 15.6% of patients showed a positive test result (Group P). These patients were then treated with a corticosteroid and an antibiotic for 2 weeks and subjected to another MMP test. Most patients (89.3%) became negative (Group P_e_) after the treatment, whereas 10.7% of patients remained positive (Group P_r_).

Figure 3 shows representative results of gelatin zymography using the supernatant from uterine flushing fluid. The previous report had implied that there was no correlation between MMP score and protein concentration in the flushing fluid of RIF [15]. Therefore all neat samples were diluted 20-folds before being run on the gels and the MMP score was defined by the intensity of each band. In this example, only lane 4 is deemed positive with totally scored 8 (≥5). Of the patients who agreed to undergo the MMP test, 15.6% (n=56) were deemed to have a positive response (Group P); the remaining 84.4% (n=304) of patients had low (≤4) MMP scores (Figure 4). After 2 weeks treatment of patients in Group P with an antibiotic and corticosteroid, 89.3% (n=50) had a negative response to the second MMP test (Group Pe). The MMP score remained high in 10.7% (n=6) of patients (Group Pr).

**Figure 5:**
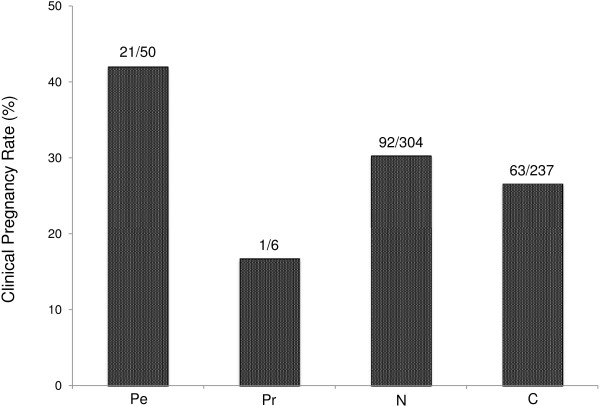
**Clinical pregnancy rate after the next embryo transfer for the four groups of RIF patients.** Group P_e_, a positive initial MMP test, but a negative second MMP test after the treatment; Group P_r_, a positive initial MMP test, and a positive second MMP test despite the treatment; Group N, a negative response to the MMP test; Group C, the control group who did not undergo the MMP test. Patients in Group P_e_ had a significantly greater pregnancy rate than patients in Groups N and C (*P < 0.05).

As indicated in Figure 5, patients in Group Pe had a higher clinical pregnancy rate than patients in Group C (21/50 [42.0%] vs 63/237 [26.6%], respectively; *P* < 0.05). In addition, patients in Group P_e_ tended to have a higher clinical pregnancy rate than patients who tested negative to the first MMP test (Group N), although the difference did not reach statistical significance. The miscarriage rates in Groups Pe, Pr, N, and C were 6/21 (28.6%), 1/1 (100%), 29/92 (31.5%), and 23/63 (36.5%), respectively. Although there was a tendency for a lower miscarriage rate in Group Pe compared with Group C patients, the difference did not reach statistical significance.

## Discussion

Numerous studies have reported on the roles of various molecules in ensuring a receptive endometrium and uterine milieu [25-29]. Some studies have used uterine flushing as a simple and minimally invasive technique to assess the cytokine concentration of uterine secretions around the time of implantation *in vivo*[15,20,24,30,31]. Lopata *et al.* used zymography to measure MMP-2 and MMP-9 activity in uterine flushing fluid and reported the band intensity of the MMPs using an “MMP score” [24]. Using this MMP scoring system, another study showed that overexpression of MMP-2 and MMP-9 is associated with increased IL-1β levels in RIF patients [15]. This previous study suggests that chronic uterine inflammation contributes to implantation failure because IL-1β is a representative inflammatory cytokine and an inducer of MMPs. In other words, chronic uterine inflammation is considered to be a major cause of RIF and high MMP activity.

The present study investigated whether treatment of an unfavorable uterine environment in women with high MMP scores could normalize MMP activity and improve rates of successful implantation. We hypothesized that antibiotic and corticosteroid treatment for the chronic inflammation responsible for MMP overactivity would reduce MMP scores in uterine flushings, improve the uterine environment for implantation, and consequently improve clinical pregnancy outcomes in patients with RIF. The results of the present study strongly support our hypothesis. After treatment, most patients in Group P (i.e. those with RIF and a positive result on the first MMP test) had a negative response to the second MMP test. Moreover, there were better clinical pregnancy rates and a tendency for a decrease in miscarriage rates in this group compared with Groups C and N.

There are some issues that require further discussion. First is the question of the effects of uterine flushings themselves on implantation. There have been some trials investigating improved pregnancy outcomes following uterine endometrial injury or uterine cavity flushing. In these studies, local injury to the endometrium, such as endometrial biopsy or curettage, is usually applied before the cycle used for embryo transfer and has been shown to improve the chances of successful implantation [32,33]. In contrast, uterine flushing or injection of medium into the uterine cavity is usually performed during the same cycle as embryo transfer [34]. The effect of simple flushing of the uterine cavity remains contentious [35,36]. In the present study, uterine flushing was performed once for Group N, but twice for Group P. Regardless of the number of uterine flushings, in all cases they occurred in cycles other than those used for embryo transfer. Therefore, we believe that any effect of the uterine flushing on embryo implantation is likely to be very limited. The second is regarding the broad effects of corticosteroids on the uterine environment. Although we used a corticosteroid to control uterine inflammation, including non-infectious endometritis, in the present study, the corticosteroid has a myriad of effects other than its anti-inflammatory actions. It has been reported that the high numbers of uterine natural killer cells (uNK) in the endometria of women with recurrent miscarriage were reduced following the administration of corticosteroids [37,38]. Because uNK cells secrete not only various cytokines, such as TNF-α, IFN-γ, TGF-β1 and IL-1β, but also MMP-2 and MMP-9 [5], the reduction in MMP scores in the present study may be due, in part, to a reduction in the number of uNK cells.

The third is the choice of a control group. If Group P_e_ is compared with Group N, there was a tendency for a higher pregnancy rate in Group P_e_ after treatment. This could be due to differences between Groups P_e_ and N in terms of the underlying cause of RIF. For example, the likely cause of RIF in MMP-positive patients is high MMP activity related to inflammation, whereas RIF in MMP-negative patients may be due to something other than inflammation. Therefore, after elimination of the cause of RIF by medication, Group P_e_ patients had a better pregnancy rate than Group N, who did not undergo any treatment for the cause of RIF. In the present study, we chose Group C as the control group. Patients in Group C did not undergo any medical intervention. In contrast, patients in Group P_e_ were identified as having high MMP activity in the setting of RIF and subsequently underwent treatment to correct high MMP activity. By comparing these two groups, our aim was to determine whether the intervention was effective in improving pregnancy rates in RIF. Strictly speaking, the present study should have included a more appropriate control group consisting of patients matched with those in Group P and treated with placebo. However, there was a large number of patients enrolled in the present study and there was clear change in the MMP test results from positive to negative after treatment in approximately 90% of patients. Therefore, we concluded the 2-week treatment period was sufficiently effective in normalizing the receptivity of the uterus.

Recently, benign endometrial pathologies have been linked to implantation failure and infertility [39-41]. Indeed, Inagaki *et al.*[20] have reported that MMP scores of patients with benign uterine complications, such as leiomyoma, adenomyosis, or endometrial polyps, are higher than in the control group. In the present study, those who have such uterine complications were excluded. However, we did undertake another investigation about RIF with uterine complications (see Additional file 1: Figure S1), which showed that the same treatment for 2 weeks was also effective to reduce the high MMP scores in the RIF patients with uterine complications. To date, the exact mechanisms by which these uterine disorders often limit fertility remain unclear, but changes in the endometrial immune and molecular environment have been suggested as a possible cause [41]. A marked increase in the density of macrophages and natural killer (NK) cells in endometrial stromal cells has been observed in the uteri of women with adenomyosis [42]. Both macrophages and uterine NK cells are known to be able to release inflammatory cytokines. Presumably these uterine disorders may bring about a condition similar to that of chronic inflammation in the endometrium, resulting in an induction of MMP activity and MMP production by endometrial stromal cells in response to the macrophages and other inflammatory cytokines [11,12].

Chronic endometritis is the most frequent inflammatory condition of the uterine endometrium that is thought to be related to infertility and spontaneous abortion [43,44]. The prevalence of chronic endometritis in the general population is estimated to range between 0.8% and 19.0% [45]. In patients undergoing ART, the prevalence of chronic endometritis has been reported to be approximately 15%, or up to 42% of patients with RIF [46,47]. Thus, there is considerable variation in the reported prevalence of chronic endometritis. This may be due to the fact that mild chronic endometritis is often clinically silent, as well as to difficulties in diagnosing the condition based on histopathological examination or using a hysteroscope [48,49]. However, the MMP test used in the present study is able to detect inflammatory conditions caused by mild and chronic endometritis. The overexpression of MMPs is induced by any type of inflammatory condition, and not just infection, hyperplasia or carcinoma, whereas the appearance of many other inflammatory cells and mediators is sometimes temporary and specifically dependent on the cause of the inflammation. As such, the MMP test can be used to detect various causes of inflammation that may create an unfavorable environment for implantation.

In the present study, only approximately 16% of all patients tested positive on the first MMP test, despite the sensitivity of the test. This rate is lower than expected based on the reported frequency of chronic endometritis in RIF patients. However, we need to take into account the advanced maternal age of the patients recruited to the present study; older patients are more likely to have embryos with chromosomal abnormalities. Indeed, it has been reported [50] that the frequency of aneuploid embryos is higher in older RIF patients (mean age 39.8 years), similar to the mean patient age of 38.1 years in the present study; specifically, the rate of aneuploidy in oocytes and blastocysts was 65.5% and 45.2%, respectively. Thus, of the possible causes of RIF, embryonic factors, particularly chromosomal abnormalities, become more important with increasing maternal age. Taking this into consideration, the fact that 16% of women were positive to the first MMP test in the present study is not necessarily low and indicates that, although not a major cause of RIF, high MMP activity is likely to contribute to a considerable number of cases of RIF. The results of the present study show that an inflammatory uterine environment can be detected by the MMP test and can be treated successfully to improve uterine receptivity.

## Conclusions

The MMP test using uterine flushing fluid and the newly designed treatment strategy of a 2-week course of antibiotics and corticosteroids is likely to be useful for women with RIF. However, further studies, including prospective studies, are needed to clarify a causal relationship between the medication and pregnancy outcomes. In addition, the routine use of a corticosteroid is not recommended before further studies have been conducted to confirm its effect against uterine non-infectious inflammation in RIF.

## Abbreviations

MMP: Matrix metalloproteinase; RIF: Recurrent implantation failure

## Competing interests

The authors declare that they have no competing interests.

## Authors’ contributions

NY and YY designed the project and experiments. NY, TH, and YO took uterine flushing samples from the patients. MMP zymography and scoring were carried out by OI, MY, RM, and NI. NY and TH wrote the paper. TH and YY organized and supervised the project. All authors read and approved the final manuscript.

## Supplementary Material

Additional file 1: Figure S1 Results of the matrix metalloproteinase (MMP) test for recurrent implantation failure (RIF) patients with mild uterine complications. Click here for file

## References

[B1] Lee TH Chen CD Tsai YY Chang LJ Ho HN Yang YS Embryo quality is more important for younger women whereas age is more important for older women with regard to in vitro fertilization outcome and multiple pregnancy Fertil Steril 2006 86 64 69 16716314 10.1016/j.fertnstert.2005.11.074

[B2] Navot D Bergh PA Williams MA Garrisi GJ Guzman I Sandler B Grunfeld L Poor oocyte quality rather than implantation failure as a cause of age-related decline in female fertility Lancet 1991 337 1375 1377 1674764 10.1016/0140-6736(91)93060-m

[B3] Déchaud H Maudelonde T Daurès JP Rossi JF Hédon B Evaluation of endometrial inflammation by quantification of macrophages, T lymphocytes, and interleukin-1 and −6 in human endometrium J Assist Reprod Genet 1998 15 612 618 9866071 10.1023/A:1020337528607 PMC3454861

[B4] Mukherjee T Copperman AB McCaffrey C Cook CA Bustillo M Obasaju MF Hydrosalpinx fluid has embryotoxic effects on murine embryogenesis: a case for prophylactic salpingectomy Fertil Steril 1996 66 851 853 8893701 10.1016/s0015-0282(16)58652-x

[B5] Strandell A Waldenström U Nilsson L Hamberger L Hydrosalpinx reduces in-vitro fertilization/embryo transfer pregnancy rates Hum Reprod 1994 9 861 863 7929732 10.1093/oxfordjournals.humrep.a138606

[B6] Vandromme J Chasse E Lejeune B Van Rysselberge M Delvigne A Leroy F Hydrosalpinges in in-vitro fertilization: an unfavourable prognostic feature Hum Reprod 1995 10 576 579 7782435 10.1093/oxfordjournals.humrep.a135992

[B7] Woessner JF Jr Matrix metalloproteinases and their inhibitors in connective tissue remodeling FASEB J 1991 5 2145 2154 1850705

[B8] Salamonsen LA Woolley DE Matrix metalloproteinases in normal menstruation Hum Reprod 1996 Suppl 2 124 133 8982754 10.1093/humrep/11.suppl_2.124

[B9] Shah BH Catt KJ Matrix metalloproteinases in reproductive endocrinology Trends Endocrinol Metab 2004 15 47 49 15080147 10.1016/j.tem.2004.01.004

[B10] Zhang J Nie G Jian W Woolley DE Salamonsen LA Mast cell regulation of human endometrial matrix metalloproteinases: a mechanism underlying menstruation Biol Reprod 1998 59 693 703 9716571 10.1095/biolreprod59.3.693

[B11] Huang HY Wen Y Irwin JC Kruessel JS Soong YK Polan ML Cytokine-mediated regulation of 92-kilodalton type IV collagenase, tissue inhibitor or metalloproteinase-1 (TIMP-1), and TIMP-3 messenger ribonucleic acid expression in human endometrial stromal cells J Clin Endocrinol Metab 1998 83 1721 1729 9589682 10.1210/jcem.83.5.4810

[B12] Salamonsen LA Butt AR Hammond FR Garcia S Zhang J Production of endometrial matrix metalloproteinases, but not their tissue inhibitors, is modulated by progesterone withdrawal in an in vitro model for menstruation J Clin Endocrinol Metab 1997 82 1409 1415 9141525 10.1210/jcem.82.5.3920

[B13] Liu G Zhang X Lin H Wang H Li Q Ni J Zhu C Effects of E-cadherin on mouse embryo implantation and expression of matrix metalloproteinase-2 and -9 Biochem Biophys Res Commun 2006 343 832 838 16564031 10.1016/j.bbrc.2006.02.189

[B14] Mulayim N Savlu A Guzeloglu-Kayisli O Kayisli UA Arici A Regulation of endometrial stromal cell matrix metalloproteinase activity and invasiveness by interleukin-8 Fertil Steril 2004 81 Suppl 1 904 911 15019828 10.1016/j.fertnstert.2003.11.015

[B15] Inagaki N Stern C McBain J Lopata A Kornman L Wilkinson D Analysis of intra-uterine cytokine concentration and matrix-metalloproteinase activity in women with recurrent failed embryo transfer Hum Reprod 2003 18 608 615 12615834 10.1093/humrep/deg139

[B16] Rinehart J Recurrent implantation failure: definition J Assist Reprod Genet 2007 24 284 287 17674185 10.1007/s10815-007-9147-4 PMC3455006

[B17] Thornhill AR deDie-Smulders CE Geraedts JP Harper JC Harton GL Lavery SA Moutou C Robinson MD Schmutzler AG Scriven PN Sermon KD Wilton L ESHRE PGD consortium 'Best practice guidelines for clinical preimplantation genetic diagnosis (PGD) and preimplantation genetic screening (PGS)' Hum Reprod 2005 20 35 48 15539444 10.1093/humrep/deh579

[B18] Gardner DK Lane M Stevens J Schlenker T Schoolcraft WB Blastocyst score affects implantation and pregnancy outcome: towards a single blastocyst transfer Fertil Steril 2000 73 1155 1158 10856474 10.1016/s0015-0282(00)00518-5

[B19] Veeck L Veeck L **Preembryo grading and degree of cytoplasmic fragmentation** An atlas of human gametes and conceptuses: an illustrated reference for assisted reproductive technology 1999 New York, USA: Parthenon 46 51

[B20] Inagaki N Ung L Otani T Wilkinson D Lopata A Uterine cavity matrix metalloproteinases and cytokines in patients with leiomyoma, adenomyosis or endometrial polyp Eur J Obstet Gynecol Reprod Biol 2003 111 197 203 14597251 10.1016/s0301-2115(03)00244-6

[B21] Kleiner DE Stetler-Stevenson WG Quantitative zymography: detection of picogram quantities of gelatinases Anal Biochem 1994 218 325 329 8074288 10.1006/abio.1994.1186

[B22] Mikolajczyk M Wirstlein P Skrzypczak J Leukaemia inhibitory factor and interleukin 11 levels in uterine flushings of infertile patients with endometriosis Hum Reprod 2006 21 3054 3058 17000646 10.1093/humrep/del225

[B23] Fridman R Fuerst TR Bird RE Hoyhtya M Oelkuct M Kraus S Komarek D Liotta LA Berman ML Stetler-Stevenson WG Domain structure of human 72-kDa gelatinase/type IV collagenase. Characterization of proteolytic activity and identification of the tissue inhibitor of metalloproteinase-2 (TIMP-2) binding regions J Biol Chem 1992 267 15398 15405 1322396

[B24] Lopata A Agresta F Quinn MA Smith C Ostor AG Salamonsen LA Detection of endometirial cancer by determination of matrix metalloproteinases in the uterine cavity Gynecol Oncol 2003 90 318 324 12893193 10.1016/s0090-8258(03)00328-7

[B25] Inoue T Kanzaki H Iwai M Imai K Narukawa S Higuchi T Katsuragawa H Mori T Tumour necrosis factor alpha inhibits in-vitro decidualization of human endometrial stromal cells Hum Reprod 1994 9 2411 2417 7714166 10.1093/oxfordjournals.humrep.a138460

[B26] Kariya M Kanzaki H Takakura K Imai K Okamoto N Emi N Kariya Y Mori T Interleukin-1 inhibits in vitro decidualization of human endometrial stromal cells J Clin Endocrinol Metab 1991 73 1170 1174 1955497 10.1210/jcem-73-6-1170

[B27] Kojima K Kanzaki H Iwai M Hatayama H Fujimoto M Inoue T Horie K Nakayama H Fujita J Mori T Expression of leukemia inhibitory factor in human endometrium and placenta Biol Reprod 1994 50 882 887 7515287 10.1095/biolreprod50.4.882

[B28] Lessey BA Castelbaum AJ Sawin SW Sun J Integrins as markers of uterine receptivity in women with primary unexplained infertility Fertil Steril 1995 63 535 542 7851583

[B29] Song H Lim H Paria BC Matsumoto H Swift LL Morrow J Bonventre JV Dey SK Cytosolic phospholipase A2alpha is crucial [correction of A2alpha deficiency is crucial] for 'on-time' embryo implantation that directs subsequent development Development 2002 129 2879 2889 12050136 10.1242/dev.129.12.2879

[B30] Licht P Lösch A Dittrich R Neuwinger J Siebzehnrübl E Wildt L Novel insights into human endometrial paracrinology and embryo-maternal communication by intrauterine microdialysis Hum Reprod Update 1998 4 532 538 10027606 10.1093/humupd/4.5.532

[B31] Mikołajczyk M Skrzypczak J Szymanowski K Wirstlein P The assessment of LIF in uterine flushing–a possible new diagnostic tool in states of impaired fertility Reprod Biol 2003 3 259 270 14688825

[B32] Nastri CO Gibreel A Raine-Fenning N Maheshwari A Ferriani RA Bhattacharya S Martins WP Endometrial injury in women undergoing assisted reproductive techniques Cochrane Database Syst Rev 2012 7 CD009517(1-43) 22786529 10.1002/14651858.CD009517.pub2

[B33] Shohayeb A El-Khayat W Does a single endometrial biopsy regimen (S-EBR) improve ICSI outcome in patients with repeated implantation failure? a randomised controlled trial Eur J Obstet Gynecol Reprod Biol 2012 164 176 179 22835632 10.1016/j.ejogrb.2012.06.029

[B34] Goto S Kadowaki T Hashimoto H Kokeguchi S Shiotani M Stimulation of endometrium embryo transfer can improve implantation and pregnancy rates for patients undergoing assisted reproductive technology for the first time with a high-grade blastocyst Fertil Steril 2009 92 1264 1268 18930200 10.1016/j.fertnstert.2008.08.076

[B35] Olivennes F Lédée-Bataille N Samama M Kadoch J Taupin JL Dubanchet S Chaouat G Frydman R Assessment of leukemia inhibitory factor levels by uterine flushing at the time of egg retrieval does not adversely affect pregnancy rates with in vitro fertilization Fertil Steril 2003 79 900 904 12749427 10.1016/s0015-0282(02)04949-x

[B36] Berkkanoglu M Isikoglu M Seleker M Ozgur K Flushing the endometrium prior to the embryo transfer does not affect the pregnancy rate Reprod Biomed Online 2006 3 268 271 16895645 10.1016/s1472-6483(10)60625-6

[B37] Quenby S Kalumbi C Bates M Farquharson R Vince G Prednisolone reduces preconceptual endometrial natural killer cells in women with recurrent miscarriage Fertil Steril 2005 84 980 984 16213853 10.1016/j.fertnstert.2005.05.012

[B38] Quenby S Nik H Innes B Lash G Turner1 M Drury J Bulmer J Uterine natural killer cells and angiogenesis in recurrent reproductive failure Hum Reprod 2009 24 45 54 18835875 10.1093/humrep/den348

[B39] Check JH Choe JK Lee G Dietterich C The effect on IVF outcome of small intramural fibroids not compressing the uterine cavity as determined by a prospective matched control study Hum Reprod 2002 17 1244 1248 11980746 10.1093/humrep/17.5.1244

[B40] Hart R Khalaf Y Yeong CT Seed P Taylor A Braude P A prospective controlled study of the effect of intramural uterine fibroids on the outcome of assisted conception Hum Reprod 2001 16 2411 2417 11679530 10.1093/humrep/16.11.2411

[B41] Rackow BW Jorgensen E Taylor HS Endometrial polyps affect uterine receptivity Fertil Steril 2011 95 2690 2692 21269620 10.1016/j.fertnstert.2010.12.034 PMC3096716

[B42] Tremellen KP Russell P The distribution of immune cells and macrophages in the endometrium of women with recurrent reproductive failure. II: adenomyosis and macrophages J Reprod Immunol 2012 93 58 63 22209314 10.1016/j.jri.2011.12.001

[B43] Kitaya K Prevalence of chronic endometritis in recurrent miscarriages Fertil Steril 2011 95 1156 1158 21030015 10.1016/j.fertnstert.2010.09.061

[B44] Romero R Espinoza J Mazor M Can endometrial infection/inflammation explain implantation failure, spontaneous abortion, and preterm birth after in vitro fertilization? Fertil Steril 2004 82 799 804 15482749 10.1016/j.fertnstert.2004.05.076

[B45] Farooki MA Epidemiology and pathology of chronic endometritis Int Surg 1967 48 566 573 6064728

[B46] Kasius JC Fatemi HM Bourgain C Sie-Go DM Eijkemans RJ Fauser BC Devroey P Broekmans FJ The impact of chronic endometritis on reproductive outcome Fertil Steril 2011 96 1451 1456 22019126 10.1016/j.fertnstert.2011.09.039

[B47] Johnston-MacAnanny EB Hartnett J Engmann LL Nulsen JC Sanders MM Benadiva CA Chronic endometritis is a frequent finding in women with recurrent implantation failure after in vitro fertilization Fertil Steril 2010 93 437 441 19217098 10.1016/j.fertnstert.2008.12.131

[B48] Kitaya K Tada Y Taguchi S Funabiki M Hayashi T Nakamura Y Local mononuclear cell infiltrates in infertile patients with endometrial macropolyps versus micropolyps Hum Reprod 2012 Epub ahead of print 10.1093/humrep/des323 22951914

[B49] Polisseni F Bambirra EA Camargos AF Detection of chronic endometritis by diagnostic hysteroscopy in asymptomatic infertile patients Gynecol Obstet Invest 2003 55 205 210 12904693 10.1159/000072075

[B50] Fragouli E Katz-Jaffe M Alfarawati S Stevens J Colls P Goodall NN Tormasi S Gutierrez-Mateo C Prates R Schoolcraft WB Munne S Wells D Comprehensive chromosome screening of polar bodies and blastocysts from couples experiencing repeated implantation failure Fertil Steril 2010 94 875 887 19540479 10.1016/j.fertnstert.2009.04.053

