# Supplementary material for: Successful implantation after reducing matrix metalloproteinase activity in the uterine cavity
Source: Reprod Biol Endocrinol. 2013 May 11;11:37. doi: 10.1186/1477-7827-11-37 (PMC3655829; doi:10.1186/1477-7827-11-37)
Supplement: Additional file 1: Figure S1 — Results of the matrix metalloproteinase (MMP) test for recurrent implantation failure (RIF) patients with mild uterine complications. [file 1477-7827-11-37-S1.pdf]

Additional file 1: Figure S1

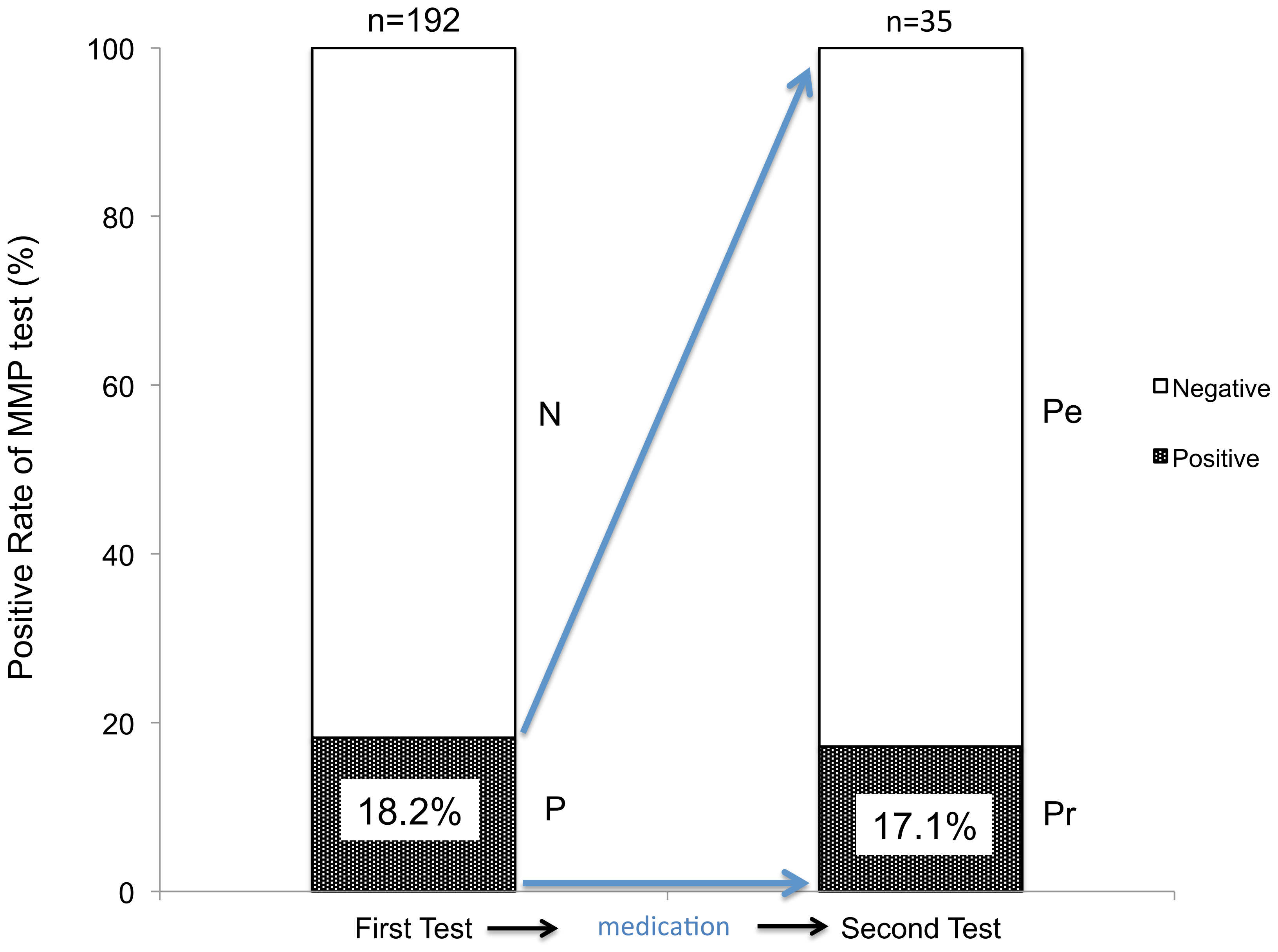

Additional file 1

Figure S1: Results of the matrix metalloproteinase (MMP) test for recurrent implantation failure (RIF) patients with mild uterine complications.

File format: PDF

Description of data

The main study excluded RIF patients with uterine complications, such as leiomyoma, adenomyosis, or endometrial polyps. (For patients with severe complications that are obviously deleterious to fertility, we recommend surgical resection or medical treatment prior to embryo transfer.) However, we did undertake analysis of MMP levels in patients with RIF who had complications that were either silent or not so severe to determine whether medical treatment could reduce MMP levels in these patients with organic complications. The results of those studies are reported herein. Ultrasound scanning, magnetic resonance imaging, and hysterofiberscopy revealed over 300 RIF patients with some form of benign uterine complication without symptoms; of these, 192 patients agreed to undergo the MMP test. As indicated in Figure S1, 35 (18.2%) of the 192 patients had a positive result on the MMP test. The 35 patients who were positive on the first MMP test underwent 2 weeks treatment with a quinolone antibiotic and a corticosteroid before undergoing a second MMP test. As shown in Figure S1, after 2 weeks treatment 82.9% of RIF patients with mild uterine complications were negative on the second test (Group P_e), with only 17.1% of patients remaining positive (Group P_r).
